# Net Clinical Benefit of Direct Oral Anticoagulants in Patients With Cancer and Venous Thromboembolism: A Systematic Review and Trade-Off Analysis

**DOI:** 10.3389/fcvm.2020.586020

**Published:** 2020-11-12

**Authors:** Yi-Dan Yan, Zheng Ding, Mang-Mang Pan, Qing Xia, Jiu-Jie Cui, Li-Wei Wang, Chi Zhang, Zhi-Chun Gu

**Affiliations:** ^1^Department of Pharmacy, School of Medicine, Renji Hospital, Shanghai Jiaotong University, Shanghai, China; ^2^Department of Pharmacy, Fuwai Hospital, Chinese Academy of Medical Sciences, Beijing, China; ^3^Department of Oncology, School of Medicine, Renji Hospital, Shanghai Jiaotong University, Shanghai, China

**Keywords:** direct oral anticoagulants (DOACs), cancer, venous thromboembolism (VTE), trade-off analysis, net clinical benefit (NCB)

## Abstract

**Background:** Venous thromboembolism (VTE) is highly prevalent in cancer patients. Recent guidelines recommend considering direct oral anticoagulants (DOACs) for the treatment of cancer-associated thrombosis (CAT). However, direct head-to-head comparisons among DOACs are lacking, and almost no net clinical benefit (NCB) analysis has been performed in patients with CAT.

**Methods:** We systematically searched PubMed, EMBASE, Cochrane Library, and ClinicalTrials.gov for randomized controlled trials (RCTs) reporting on recurrent VTE, major bleeding, or clinically relevant bleeding events in patients with CAT who received DOACs and low-molecular-weight heparins. Relative risks (RRs) and 95% confidence intervals (95% CIs) were calculated using a random-effect model. Surface under the cumulative ranking curve (SUCRA) values were calculated, and a trade-off analysis was performed to estimate the NCB.

**Results:** Overall, four RCTs involving 2,894 patients were enrolled. DOACs were more effective than dalteparin in reducing the risk of recurrent VTE (RR: 0.62, 95% CI: 0.44–0.87), with a comparative risk of major bleeding (RR: 1.33, 95% CI: 0.84–2.11) and an increased risk of clinically relevant bleeding (RR: 1.45, 95% CI: 1.05–1.99). No significant difference was observed among individual anticoagulants in terms of recurrent VTE and major bleeding. With respect to the ranking of each anticoagulant for the primary outcome, edoxaban (SUCRA: 69.2) was more effective than dalteparin (SUCRA: 60.7), rivaroxaban (SUCRA: 60.7), and apixaban (SUCRA: 25.5) in reducing VTE recurrence. For major bleeding, apixaban (SUCRA: 76.3) had the highest cumulative ranking probability, followed by edoxaban (SUCRA: 66.4), dalteparin (SUCRA: 28.8), and rivaroxaban (SUCRA: 28.5). Similar results were observed for clinically relevant bleeding. In terms of both benefit and safety outcomes, DOACs, especially edoxaban, seemed to confer a better NCB profile than dalteparin.

**Conclusions:** DOACs are a safe and effective alternative therapy to dalteparin in patients with CAT. Among them, edoxaban might provide a good risk-to-benefit balance. However, because of the lack of head-to-head studies, further investigations are needed to confirm our findings.

## Introduction

Venous thromboembolism (VTE), including deep vein thrombosis and pulmonary embolism, is a well-recognized complication and a common cause of death in cancer patients, with an estimated incidence of 1–20% (1). Even in the presence of anticoagulation, cancer-associated thrombosis (CAT) has been associated with an approximate 10–20% risk of recurrent VTE and a 10% risk of bleeding annually, placing an enormous burden on global healthcare systems (2). Therefore, the optimal anticoagulation strategy that can balance the risks of VTE recurrence and bleeding has been the focus in this vulnerable population.

Most practice guidelines recommend low-molecular-weight heparins (LMWHs) for both prophylaxis and treatment in patients with CAT, and the importance of direct oral anticoagulants (DOACs) has recently been raised (3, 4). In previous randomized controlled trials (RCTs), namely SELECT-D and Hokusai VTE Cancer, rivaroxaban and edoxaban significantly reduced the risk of recurrent VTE; however, this benefit was partly offset by a higher risk of bleeding than with LMWHs, which inevitably limits the clinical application of these DOACs for CAT treatment (5, 6). Notably, two up-to-date trials (ADAM VTE and Caravaggio) comparing apixaban with dalteparin suggested the non-inferiority of apixaban in terms of recurrent VTE and bleeding in the treatment of CAT (7, 8). The net clinical benefit (NCB), which balances the risks of thromboembolism and hemorrhage, is crucial for identifying the optimal anticoagulation in CAT. Because head-to-head comparisons among DOACs are lacking, an indirect analysis could be performed to obtain the estimates of comparative results. In the present study, we conducted a systematic review and trade-off analysis of the efficacy and safety of DOACs to evaluate their NCB in patients with cancer and VTE.

## Methods

### Literature Search

This systematic review was conducted in line with the standards in the PRISMA (Preferred Reporting Items for Systematic Reviews and Meta-analyses) and Cochrane Collaboration Statements (9, 10). The PubMed, EMBASE, and Cochrane Library databases were systematically searched from inception to May 15, 2020, with language restricted to English. The full details of the search strategies are presented in Supplementary Table 1. Moreover, the ClinicalTrials.gov website was searched for unpublished data. Two reviewers (Y-DY and M-MP) independently assessed the titles and abstracts to determine study eligibility, and full articles were retrieved and assessed according to the inclusion criteria. Any disagreements were resolved by the corresponding author (Z-CG).

### Study Selection

The eligibility criteria for studies were as follows: (1) RCT design, (2) inclusion of patients with cancer and VTE, (3) comparison of DOACs with LMWHs, and (4) available data of outcomes including recurrent VTE and bleeding events. Studies published only in the form of a conference abstract or letter were excluded. Studies were also excluded if they involved patients with cancer and atrial fibrillation, or any other condition requiring long-term antithrombotic therapy. To determine study eligibility, two authors (Y-DY and M-MP) independently reviewed all titles and abstracts, and all papers were assessed based on entry criteria. Discrepancies were resolved through a discussion with the corresponding author (Z-CG).

### Study Outcomes

The primary efficacy outcome was the recurrence of VTE including deep vein thrombosis and pulmonary embolism. The primary safety outcomes were major bleeding and clinically relevant bleeding, as defined in each study (Supplementary Table 2).

### Data Extraction and Quality Evaluation

Data were extracted into a pre-specified form: including study characteristics (study name, number of patients, intervention, and follow-up duration); patient demographics (age, sex, renal function, proportion of cancer types); and information (recurrent VTE, major bleeding, and clinically relevant bleeding). The methodological quality of the included RCTs was evaluated using the Cochrane Collaboration Risk of Bias Tool (11).

### Statistical Analysis

Results were reported as relative risks (RRs) with 95% confidence intervals (95% CIs) using a random-effect model. Statistical heterogeneity was assessed using the *I*^2^*-*test, with a value of >50% representing considerable heterogeneity (12). In the indirect comparison among DOACs, LMWHs were used as the reference comparator. Surface under the cumulative ranking curves (SUCRA) values were calculated to rank treatments with respect to different outcomes based on cumulative probability plots, with a larger SUCRA value indicating better ranking of the treatment (13). For the NCB, clustered ranking plots of treatments were depicted according to the SUCRA probabilities for both efficacy and safety. Narrow NCB weighted both recurrent VTE and major bleeding, and broad NCB balanced recurrent VTE and clinically relevant bleeding. Publication bias was qualitatively evaluated using funnel plots when >10 studies were available in a single analysis (9). All statistical analyses were performed using the STATA software (version 13.0, STATA Corporation), with a *P*-value of <0.05 indicating statistical significance.

## Results

### Study Selection and Characteristics of the Included Studies

**Figure 1:**
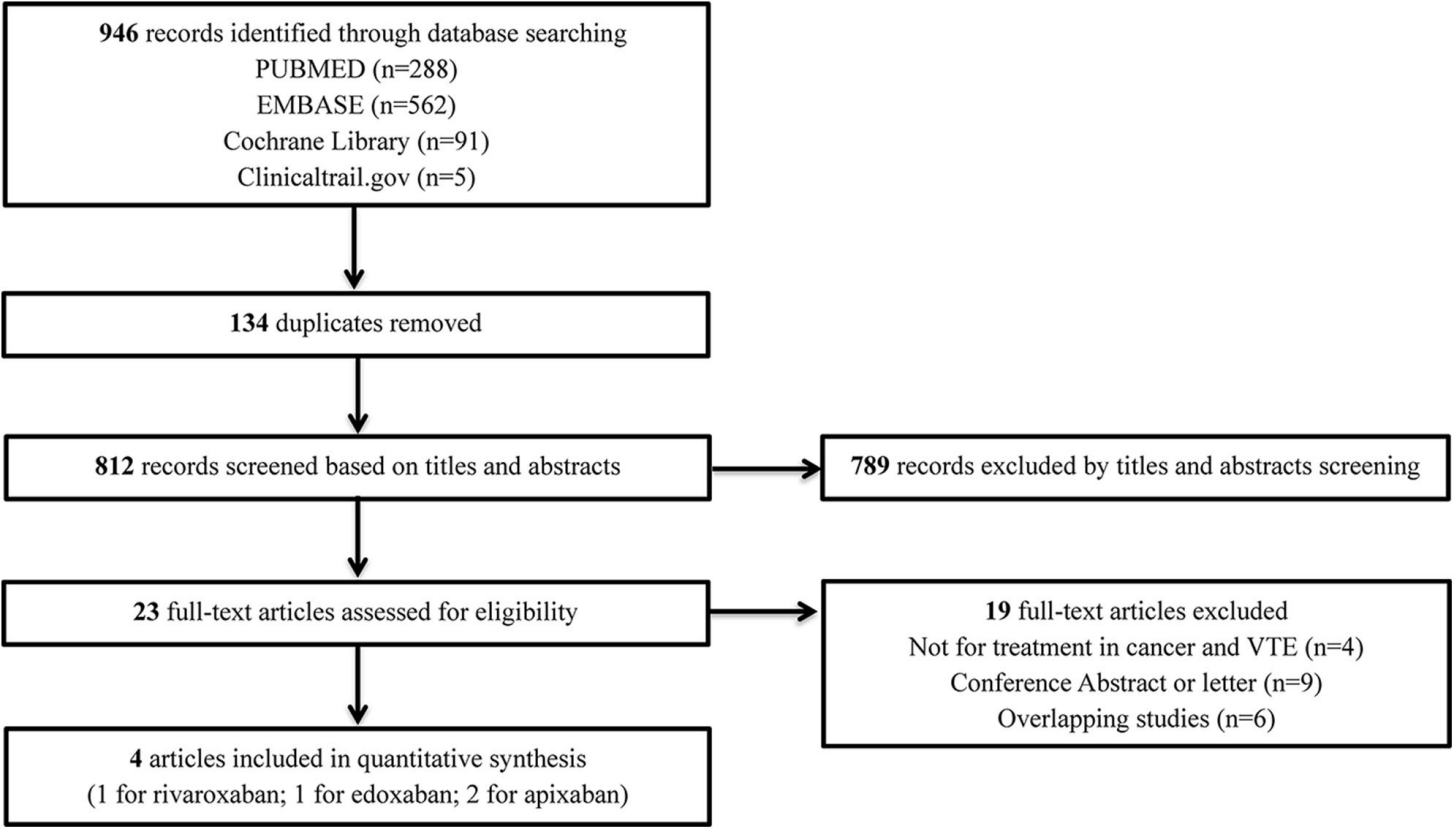
Flow diagram for the selection of eligible studies. VTE, venous thromboembolism.

**Figure 2:**
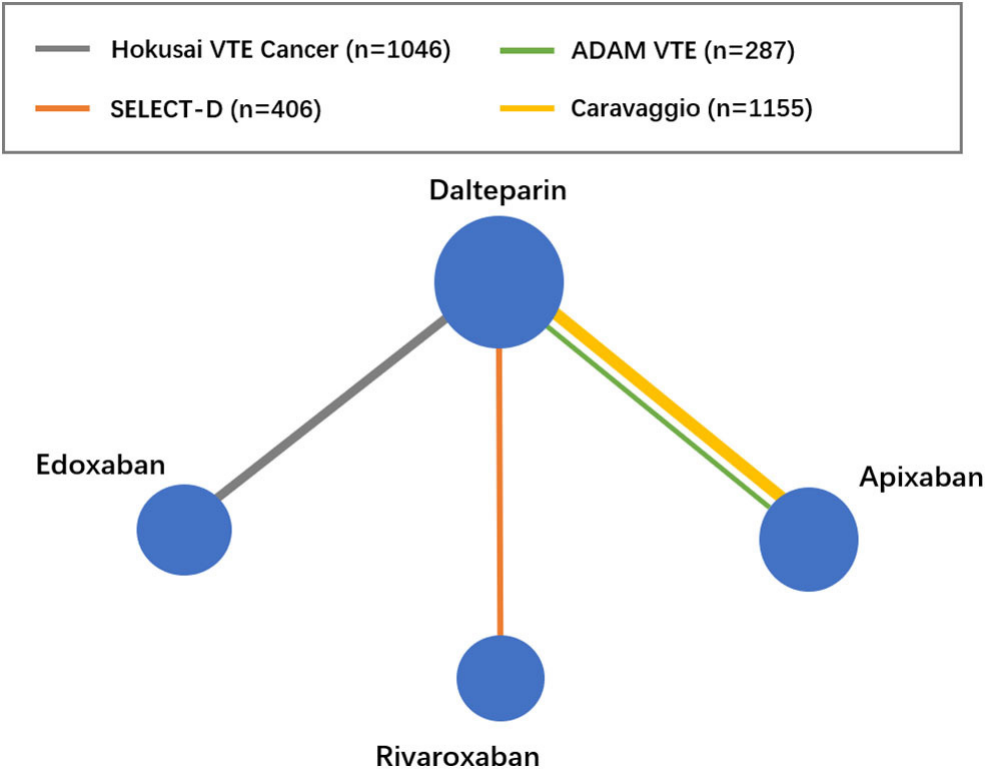
Evidence network map between DOACs and dalteparin. VTE, venous thromboembolism.

The flow diagram of the study selection process is shown in Figure 1. The initial search yielded 946 records, among which 23 full-text articles were obtained for further assessment for eligibility. Finally, four eligible RCTs (5–8) involving 2,894 patients with CAT were enrolled, with 1,446 patients using DOACs and 1,448 patients using dalteparin. The characteristics of the included studies are presented in Table 1. The mean or median age of the patients ranged from 64 to 67 years, and the proportion of women ranged from 47 to 52%. The tumor types were comparable across the four trials, with the exception of gastric (2.5–5.2%), gynecologic (3.0–10.5%), and hematologic (2.5–10.6%) cancers. An evidence network map comparing DOACs and dalteparin is outlined in Figure 2. The overall quality of the four RCTs was relatively high (Table 1).

**Table 1:** Details of studies included in the analysis.

**Study**	**Number**	**Interventions**	**Follow-up**	**Age, yr (mean/median)**	**Female (%)**	**Ccr of 30–50 ml/min (%)**	**Metastatic cancer (%)**	**Hematologic cancer (%)**	**Colorectal cancer (%)**	**Lung cancer (%)**	**Breast cancer (%)**	**Gynecologic cancer (%)**	**Gastric cancer (%)**	**Risk of bias**
Hokusai VTE Cancer (2018)	1,046	Edoxaban 60 mg once Dalteparin	12 m	64.3	47.7	6.9	53	10.6	15.5	14.6	11.9	10.5	5.2	Low
SELECT-D (2018)	406	Rivaroxaban 15 mg twice and then 20 mg once Dalteparin	6 m	67	47	NR	58	2.5	25	11.5	10	3	2.5	Low
ADAM VTE (2019)	287	Apixaban 10 mg twice and then 5 mg twice Dalteparin	6 m	64.4	51.7	9.3	65.7	9.3	15.9	17.3	9	9.8	3.8	Low
Caravaggio (2020)	1,155	Apixaban 10 mg twice and then 5 mg twice Dalteparin	6 m	67.2	50.8	9.7	NR	7.4	20.3	17.3	13.4	10.3	4.7	Low

*VTE, venous thromboembolism; yr, year; m, month; Ccr, creatinine clearance rate; NR, not reported*.

### Efficacy and Safety of DOACs in Patients With CAT

Both direct and indirect comparisons were conducted for efficacy (recurrent VTE) and safety (major bleeding and clinically relevant bleeding). In the direct comparison, DOACs were more effective than dalteparin in reducing the risk of recurrent VTE (RR: 0.62, 95% CI: 0.44–0.87), with a comparative risk of major bleeding (RR: 1.33, 95% CI: 0.84–2.11) and an increased risk of clinically relevant bleeding (RR: 1.45, 95% CI: 1.05–1.99) in patients with CAT (Table 2). In the indirect comparison, no significant difference was observed among individual anticoagulants (dalteparin, edoxaban, rivaroxaban, and apixaban) in terms of recurrent VTE and major bleeding. With respect to clinically relevant bleeding, edoxaban (RR: 1.33, 95% CI: 1.01–1.76) and rivaroxaban (RR: 2.77, 95% CI: 1.51–5.06) significantly increased the risk compared with dalteparin, and rivaroxaban showed a higher risk than apixaban (RR: 2.27, 95% CI: 1.15–4.48) (Table 3).

**Table 2:** Direct comparisons of treatments for the risk of clinical outcomes.

**Treatment comparison**	**No. of studies**	**With DOAC therapy**	**With dalteparin therapy**	**Total**	**RR**	**95% CI**	***I^**2**^***
**RECURRENT VTE**							
Edoxaban vs. dalteparin	1	41/522 (7.85%)	59/524 (11.26%)	100/1,046 (9.56%)	0.70	0.48–1.02	–
Rivaroxaban vs. dalteparin	1	8/203 (3.94%)	18/203 (8.87%)	26/406 (6.40%)	0.44	0.20–1.00	–
Apixaban vs. dalteparin	2	33/721 (4.58%)	55/721 (7.63%)	88/1,442 (6.10%)	0.37	0.06–2.08	66.9%
DOACs vs. dalteparin	4	82/1,446 (5.67%)	132/1,448 (9.12%)	214/2,894 (7.39%)	0.62	0.44–0.87	24.9%
**MAJOR BLEEDING**							
Edoxaban vs. dalteparin	1	36/522 (6.90%)	21/524 (4.01%)	57/1,046 (5.45%)	1.72	1.02–2.91	–
Rivaroxaban vs. dalteparin	1	11/203 (5.42%)	6/203 (2.96%)	17/406 (4.19%)	1.83	0.69–4.86	–
Apixaban vs. dalteparin	2	22/721 (3.05%)	25/721 (3.47%)	47/1,442 (3.26%)	0.89	0.47–1.71	2.3%
DOACs vs. dalteparin	4	69/1,446 (4.77%)	52/1,448 (3.59%)	121/2,894 (4.18%)	1.33	0.84–2.11	27.0%
**CLINICALLY RELEVANT BLEEDING**							
Edoxaban vs. dalteparin	1	97/522 (18.58%)	73/524 (13.93%)	170/1,046 (16.25%)	1.33	1.01–1.76	–
Rivaroxaban vs. dalteparin	1	36/203 (17.73%)	13/203 (6.40%)	49/406 (12.07%)	2.77	1.51–5.06	–
Apixaban vs. dalteparin	2	79/721 (10.96%)	65/721 (9.02%)	144/1,442 (9.99%)	1.22	0.89–1.66	0%
DOACs vs. dalteparin	4	212/1,446 (14.66%)	151/1,448 (10.43%)	363/2,894 (12.54%)	1.45	1.05–1.99	50.3%

*DOAC, direct oral anticoagulant; LMWH, low molecular weight heparin; VTE, venous thromboembolism*.

**Table 3:** Network meta-analysis for the risk of clinical outcomes.

**Treatment comparison**	**Recurrent VTE**	**Major bleeding**	**Clinically relative bleeding**
Edoxaban vs. dalteparin	0.70 (0.08, 5.94)	1.72 (0.92, 3.21)	1.33 (1.01, 1.76)
Rivaroxaban vs. dalteparin	0.44 (0.05, 4.25)	1.83 (0.65, 5.15)	2.77 (1.51, 5.06)
Apixaban vs. dalteparin	0.37 (0.05, 2.46)	0.89 (0.11, 7.35)	1.22 (0.89, 1.66)
Edoxaban vs. rivaroxaban	1.57 (0.07, 35.30)	0.94 (0.28, 3.14)	0.48 (0.25, 0.94)
Edoxaban vs. apixaban	1.91 (0.11, 33.55)	1.92 (0.21, 17.31)	1.09 (0.72, 1.66)
Rivaroxaban vs. apixaban	1.22 (0.06, 23.34)	2.05 (0.20, 21.40)	2.27 (1.15, 4.48)

*VTE, venous thromboembolism*.

### Efficacy and Safety of DOACs Based on Ranking

The ranking of each anticoagulant is shown in Table 4. In terms of recurrent VTE, edoxaban (SUCRA: 69.2) was more effective than dalteparin (SUCRA: 60.7), rivaroxaban (SUCRA: 60.7), and apixaban (SUCRA: 25.5). With respect to major bleeding, apixaban (SUCRA: 76.3) had the highest cumulative ranking probability, followed by edoxaban (SUCRA: 66.4), dalteparin (SUCRA: 28.8), and rivaroxaban (SUCRA: 28.5). Similar results were observed for clinically relevant bleeding.

**Table 4:** SUCRA ranking of anticoagulants for clinical outcomes.

**Treatment**	**Recurrent VTE**		**Major bleeding**		**Clinically relevant bleeding**	
	**SUCRA**	**Mean rank**	**SUCRA**	**Mean rank**	**SUCRA**	**Mean rank**
Edoxaban	69.2	1.9	66.4	2.0	58.9	2.2
Rivaroxaban	60.7	2.2	28.5	3.1	0.8	4.0
Apixaban	25.5	3.2	76.3	1.7	95.7	1.1
Dalteparin	60.7	2.2	28.8	3.1	44.6	2.7

*SUCRA, the surface under the cumulative ranking curve; VTE, venous thromboembolism*.

### Trade-Off Analysis Balancing Efficacy and Safety

**Figure 3:**
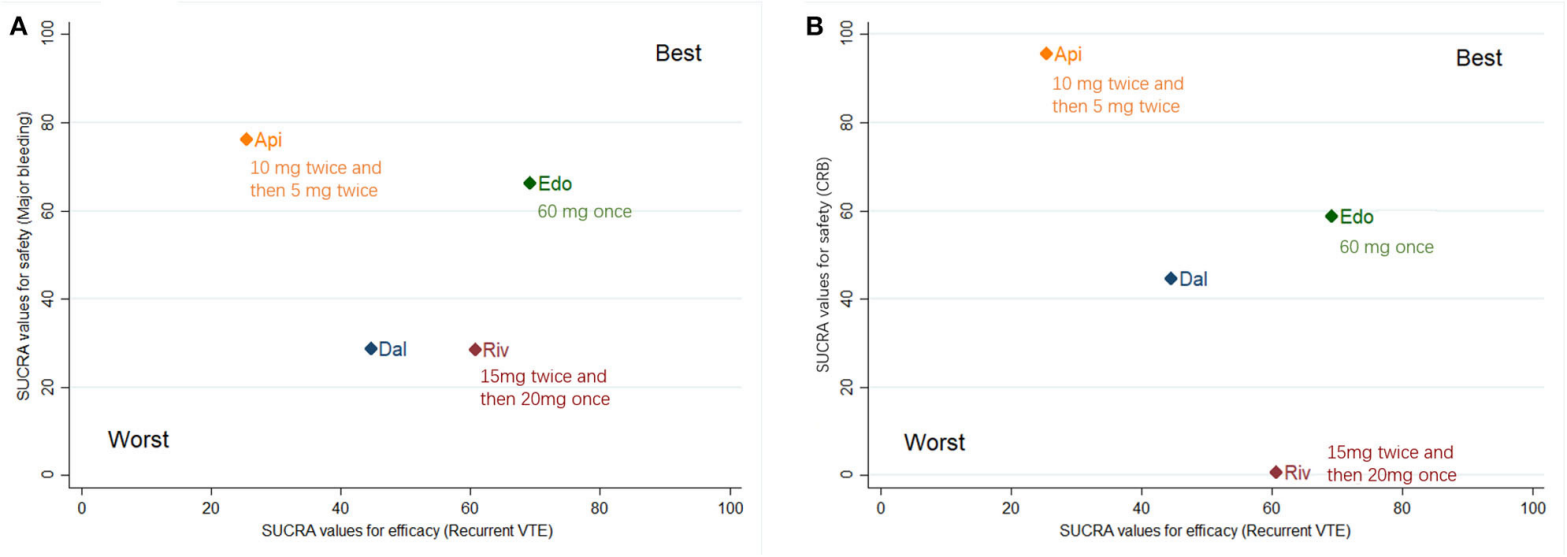
Surface under the cumulative ranking (SUCRA) plot. Ranking of treatments expresses the probability associated to each one being the best with respect to recurrent VTE and major bleeding **(A)**, as well as recurrent VTE and CRB **(B)**. Treatments in the upper right corner are more effective and safer than the other treatments. VTE, venous thromboembolism; CRB, clinically relevant bleeding; Dal, dalteparin; Edo, edoxaban; Riv, rivaroxaban; Api, apixaban.

The clustered ranking plot according to SUCRA values indicated that DOACs in general seemed to confer a better NCB profile than dalteparin, irrespective of narrow and broad NCB. Edoxaban occupied the most favorable position with respect to efficacy and safety. Apixaban formed a cluster of “relatively low effectiveness but the highest safety level,” whereas rivaroxaban presented a cluster of “moderate effectiveness and the highest bleeding risk” (Figure 3).

## Discussion

The present analysis, based on four RCTs involving 2,894 patients, demonstrated that DOACs are a safe and effective alternative therapy to dalteparin in patients with CAT. No significant difference in VTE recurrence and major bleeding was observed among individual anticoagulants in the treatment of CAT. In terms of both benefit and safety outcomes, DOACs, especially edoxaban, seemed to confer a better NCB profile than dalteparin.

Owing to hypercoagulability, patients with cancer have a high risk for both recurrent VTE, even in the presence of anticoagulation, and bleeding complications (14). Therefore, the balance between efficacy and safety is often neglected in the treatment of patients with CAT. Most guidelines recommend LMWHs as the first-line treatment for CAT, mainly based on the landmark CLOT trial (15). Two RCTs, namely Hokusai VTE Cancer and SELECT-D, provided new evidence and supported the recommendation of DOACs as an alternative to LMWHs for the treatment of CAT (5, 6). Notably, both the contemporary ADAM VTE trial and Caravaggio trial revealed that apixaban was non-inferior to dalteparin in the treatment of CAT, without increasing the risk of bleeding (7, 8), which prompted systematic reassessments of the benefits and harms associated with DOACs.

A prior meta-analysis of three RCTs (Hokusai VTE Cancer, SELECT-D, and ADAM VTE) assessed the efficacy and safety of DOACs in patients with CAT, and revealed borderline significant results for DOACs vs. dalteparin in terms of recurrent VTE (RR: 0.51, 95% CI: 0.25–1.03) and major bleeding (RR: 1.64, 95% CI: 1.00–2.69) (16). The latest Caravaggio trial (7), with a large sample size of 1,155 patients with CAT, emphasized that the use of apixaban for up to 6 months was non-inferior to subcutaneous dalteparin in terms of recurrent VTE and major bleeding. Similar to the Caravaggio trial, our results revealed that the DOACs were overall more effective than dalteparin in reducing the risk of recurrent VTE, without increasing the risk of major bleeding. However, confusion remains with respect to the optimal selection of individual DOACs because of the absence of head-to-head comparisons.

The NCB, which comprehensively considers the balance of recurrent VTE risk and bleeding risk, provides a more quantitatively informed basis for decision making. The results of our trade-off analysis indicated that DOACs may generally confer a better NCB profile than dalteparin in patients with CAT. Edoxaban was the most favorable DOAC in terms of benefit and safety. Accordingly, we speculated that edoxaban may provide a good risk-to-benefit balance. Interestingly, the Hokusai VTE Cancer trial, the only RCT that compared edoxaban with dalteparin, revealed a significantly increased risk of major bleeding with edoxaban, which was mainly due to a higher risk of upper gastrointestinal bleeding in gastric cancer patients (5). Inconsistent results between direct and indirect comparisons were observed, as in other studies (17). Unlike direct comparison, indirect analysis included both direct evidence and indirect effects from the other three studies. The smaller proportion of gastric cancer patients in the other three trials (6–8) (SELECT-D: 2.5%, ADAM VTE: 3.8%, and Caravaggio: 4.7%) than in the Hokusai VTE Cancer trial (5.2%) might explain the relatively lower bleeding risk. Such integrated results possibly underestimated the overall rate of bleeding with edoxaban compared with dalteparin and failed to demonstrate an increased risk of major bleeding. As no head-to-head RCT of DOACs exists, further studies are needed to confirm that edoxaban confers a better NCB profile in patients with CAT.

With respect to VTE prophylaxis, two emerging clinical trials (AVERT and CASSINI) compared DOACs with placebo among high-risk ambulatory cancer patients with a Khorana score of >2 (score range: 0–6, with higher scores indicating a higher risk of VTE) (18, 19). The AVERT trial revealed that apixaban therapy resulted in a significantly lower rate of VTE than placebo (HR: 0.41; 95% CI: 0.26–0.65) at the cost of an increased risk of major bleeding (HR: 2.00; 95% CI, 1.01–3.95). In contrast, the CASSINI trial indicated that rivaroxaban led to a substantially lower incidence of VTE (HR: 0.40; 95% CI: 0.20–0.80) with a low incidence of major bleeding (HR, 1.96; 95% CI: 0.59–6.49). On the basis of the above evidence, DOACs might represent an alternative choice for thromboprophylaxis in cancer patients. However, all results should be cautiously interpreted because of the absence of a direct comparison among anticoagulants.

In the clinical setting, despite fewer interactions with DOACs than with vitamin K antagonists, pharmacokinetic interactions between DOACs and anticancer drugs should be considered. Certain chemotherapeutic and targeted agents that interfere with P-glycoprotein or CYP3A4, such as vinblastine, doxorubicin, crizotinib, and sunitinib, may affect the plasma levels of DOACs and subsequently influence the anticoagulation effect (20). However, the clinical significance of drug-drug interactions between NOACs and anticancer agents is still largely unknown. Undeniably, interactions between anticancer drugs and DOACs might exist in the included studies; however, detailed information was not provided. Therefore, we did not have get access to data related to the co-administered anticancer drugs, which precluded a powerful subgroup analysis.

This study had several noteworthy limitations. First, minor differences in baseline characteristics existed across the included RCTs, especially with respect to tumor types. The overall heterogeneity among the four RCTs was moderate, as revealed by the *I*^2^*-*values. Accordingly, a random-effect model was used in the statistical analysis. Second, no statistical significance of recurrent VTE and major bleeding among the three DOACs was observed owing to an insufficient sample size in each arm of the DOACs. Further studies with direct head-to-head comparisons among DOACs are necessary. Third, we did not have access to patient-level data related to the type, stage, or location of cancer, as well as detailed information related to combination chemotherapy, which precluded a powerful subgroup analysis. In addition, publication bias could not be assessed owing to the limited number of included studies.

## Conclusions

The results of this study suggest that DOACs are a safe and effective alternative therapy to dalteparin in patients with CAT. Among the DOACs, edoxaban may provide a good risk-to-benefit balance. However, further studies with head-to-head comparisons among DOACs are needed to confirm our findings.

## Data Availability Statement

The original contributions presented in the study are included in the article/Supplementary Material, further inquiries can be directed to the corresponding authors.

## Author Contributions

Z-CG and CZ are the guarantors of the entire manuscript. Z-CG and Y-DY contributed to the study conception and design, the critical revision of the manuscript for important intellectual content, and the final approval of the version to be published. ZD, M-MP, QX, J-JC, and L-WW contributed to the data acquisition, analysis, and interpretation. All authors contributed to the article and approved the submitted version.

## Conflict of Interest

The authors declare that the research was conducted in the absence of any commercial or financial relationships that could be construed as a potential conflict of interest.

## References

[1] Ay C Pabinger I Cohen AT. Cancer-associated venous thromboembolism: burden, mechanisms, and management. Thromb Haemost. (2017) 117:219–30. 10.1160/TH16-08-0615 27882374

[2] Di Minno MND Ageno W Lupoli R Conte G van Es N Buller HR Direct oral anticoagulants for the treatment of acute venous thromboembolism in patients with cancer: a meta-analysis of randomised controlled trials. Eur Respir J. (2017) 50:1701097 10.1183/13993003.01097-2017 28954772

[3] Streiff MB Holmstrom B Angelini D Ashrani A Bockenstedt PL Chesney C. NCCN guidelines insights: cancer-associated venous thromboembolic disease, version 2.2018. J Natl Compr Canc Netw. (2018) 16:1289–303. 10.6004/jnccn.2018.0084 30442731

[4] Key NS Khorana AA Kuderer NM Bohlke K Lee AYY Arcelus JI. Venous thromboembolism prophylaxis and treatment in patients with cancer: ASCO clinical practice guideline update. J Clin Oncol. (2020) 38:496–520. 10.1200/JCO.19.01461 31381464

[5] Raskob GE van Es N Verhamme P Carrier M Di Nisio M Garcia D. Edoxaban for the treatment of cancer-associated venous thromboembolism. N Engl J Med. (2018) 378:615–24. 10.1056/NEJMoa1711948 29231094

[6] Young AM Marshall A Thirlwall J Chapman O Lokare A Hill C. Comparison of an oral factor Xa inhibitor with low molecular weight heparin in patients with cancer with venous thromboembolism: results of a randomized trial (SELECT-D). J Clin Oncol. (2018) 36:2017–23. 10.1200/JCO.2018.78.8034 29746227

[7] Agnelli G Becattini C Meyer G Munoz A Huisman MV Connors JM. Apixaban for the treatment of venous thromboembolism associated with cancer. N Engl J Med. (2020) 382:1599–607. 10.1056/NEJMoa1915103 32223112

[8] McBane RD 2nd Wysokinski WE Le-Rademacher JG Zemla T Ashrani A Tafur A. Apixaban and dalteparin in active malignancy-associated venous thromboembolism: the ADAM VTE trial. J Thromb Haemost. (2020) 18:411–21. 10.1111/jth.14662 31630479

[9] Liberati A Altman DG Tetzlaff J Mulrow C Gøtzsche PC Ioannidis JP. The PRISMA statement for reporting systematic reviews and meta-analyses of studies that evaluate health care interventions: explanation and elaboration. J Clin Epidemiol. (2009) 62:e1–34. 10.1016/j.jclinepi.2009.06.006 19631507

[10] Polzin A Dannenberg L Wolff G Zeus T Kelm M Petzold T Increased risk of myocardial infarction with dabigatran etexilate: fact or fiction? A critical meta-analysis from integrating randomized controlled trials and real-world studies: wine or spritzer? Int J Cardiol. (2018) 270:82 10.1016/j.ijcard.2018.07.020 30219540

[11] Higgins JP Altman DG Gøtzsche PC Jüni P Moher D Oxman AD. The Cochrane Collaboration's tool for assessing risk of bias in randomised trials. BMJ. (2011) 343:d5928. 10.1136/bmj.d5928 22008217 PMC3196245

[12] Gu ZC Yan YD Yang SY Shen L Kong LC Zhang C. Direct versus conventional anticoagulants for treatment of cancer associated thrombosis: a pooled and interaction analysis between observational studies and randomized clinical trials. Ann Transl Med. (2020) 8:95. 10.21037/atm.2019.12.152 32175388 PMC7049023

[13] Gu ZC Kong LC Yang SF Wei AH Wang N Ding Z. Net clinical benefit of non-vitamin K antagonist oral anticoagulants in atrial fibrillation and chronic kidney disease: a trade-off analysis from four phase III clinical trials. Cardiovasc Diagn Ther. (2019) 9:410–9. 10.21037/cdt.2019.07.09 31737513 PMC6837910

[14] Yan YD Zhang C Shen L Su YJ Liu XY Wang LW. Net clinical benefit of non-vitamin K antagonist oral anticoagulants for venous thromboembolism prophylaxis in patients with cancer: a systematic review and trade-off analysis from 9 randomized controlled trials. Front Pharmacol. (2018) 9:575. 10.3389/fphar.2018.00575 29946255 PMC6005885

[15] Lee AY Levine MN Baker RI Bowden C Kakkar AK Prins M. Low-molecular-weight heparin versus a coumarin for the prevention of recurrent venous thromboembolism in patients with cancer. N Engl J Med. (2003) 349:146–53. 10.1056/NEJMoa025313 12853587

[16] Mai V Tanguay VF Guay CA Bertoletti L Magnan S Turgeon AF. DOAC compared to LMWH in the treatment of cancer related-venous thromboembolism: a systematic review and meta-analysis. J Thromb Thrombolysis. (2020) 50:661–7. 10.1007/s11239-020-02055-1 32052314

[17] Rossel A Robert-Ebadi H Combescure C Grosgurin O Stirnemann J Addeo A. Anticoagulant therapy for acute venous thrombo-embolism in cancer patients: a systematic review and network meta-analysis. PLoS ONE. (2019) 14:e0213940. 10.1371/journal.pone.0213940 30897142 PMC6428324

[18] Carrier M Abou-Nassar K Mallick R Tagalakis V Shivakumar S Schattner A. Apixaban to prevent venous thromboembolism in patients with cancer. N Engl J Med. (2019) 380:711–9. 10.1056/NEJMoa1814468 30511879

[19] Khorana AA Soff GA Kakkar AK Vadhan-Raj S Riess H Wun T. Rivaroxaban for thromboprophylaxis in high-risk ambulatory patients with cancer. N Engl J Med. (2019) 380:720–8. 10.1056/NEJMoa1814630 30786186

[20] Steffel J Verhamme P Potpara TS Albaladejo P Antz M Desteghe L. The 2018 European Heart Rhythm Association Practical Guide on the use of non-vitamin K antagonist oral anticoagulants in patients with atrial fibrillation. Eur Heart J. (2018) 39:1330–93. 10.1093/eurheartj/ehy136 29562325

