# Supplementary material for: Net Clinical Benefit of Direct Oral Anticoagulants in Patients With Cancer and Venous Thromboembolism: A Systematic Review and Trade-Off Analysis
Source: Front Cardiovasc Med. 2020 Nov 12;7:586020. doi: 10.3389/fcvm.2020.586020 (PMC7693545; doi:10.3389/fcvm.2020.586020)
Supplement: Supplementary file 1 [file Data_Sheet_1.PDF]

Supplementary Table 1. Search strategy used in May 15, 2020

Literature databases	Search items	Items found
PUBMED	"dabigatran"[MeSH Terms] OR "dabigatran"[Title/Abstract] OR "Pradaxa"[Title/Abstract] OR "rivaroxaban"[MeSH Terms] OR "rivaroxaban"[Title/Abstract] OR "Xarelto"[Title/Abstract] OR "apixaban" [MeSH Terms] OR "apixaban"[Title/Abstract] OR "Eliquis"[Title/Abstract] OR "edoxaban"[MeSH Terms] OR "edoxaban"[Title/Abstract] OR "Savaysa"[Title/Abstract]) OR "betrixaban"[MeSH Terms] OR "betrixaban"[Title/Abstract] OR "Bevyxxa"[Title/Abstract]) OR "Non-vitamin K antagonist oral anticoagulants"[Title/Abstract] OR "NOACs"[Title/Abstract]) OR "direct oral anticoagulants"[Title/Abstract]) OR "DOACs"[Title/Abstract]) OR "novel oral anticoagulants"[Title/Abstract]) OR "new oral anticoagulants"[Title/Abstract]) OR "factor Xa inhibitors"[Title/Abstract]) OR "factor IIa inhibitors"[Title/Abstract]<br>AND<br>"cancer"[MeSH Terms] OR "cancer"[Title/Abstract] OR "neoplasia"[Title/Abstract] OR "neoplasm"[Title/Abstract] OR "tumor"[Title/Abstract] OR "malignancy"[Title/Abstract]<br>AND<br>"low molecular weight heparin" [Title/Abstract] OR "LMWH" [Title/Abstract] OR "enoxaparin" [Title/Abstract] OR "dalteparin" [Title/Abstract] OR "nadroparin" [Title/Abstract]	288

EMBASE	‘dabigatran’/exp OR ‘dabigatran’:ti,ab,kw OR ‘Pradaxa’: ti,ab,kw OR ‘rivaroxaban’/exp OR ‘rivaroxaban’: ti,ab,kw OR ‘Xarelto’: ti,ab,kw OR ‘apixaban’/exp OR ‘apixaban’: ti,ab,kw OR ‘Eliquis’: ti,ab,kw OR edoxaban’/exp OR ‘edoxaban’: ti,ab,kw OR ‘Savaysa’: ti,ab,kw OR ‘betrixaban’/exp OR ‘betrixaban’: ti,ab,kw OR ‘Bevyxxa’: ti,ab,kw OR ‘Non-vitamin K antagonist oral anticoagulants’: ti,ab,kw OR ‘NOACs’: ti,ab,kw OR ‘direct oral anticoagulants’: ti,ab,kw OR ‘DOACs’: ti,ab,kw OR ‘novel oral anticoagulants’: ti,ab,kw OR ‘new oral anticoagulants’: ti,ab,kw OR ‘factor Xa inhibitors’: ti,ab,kw OR ‘factor IIa inhibitors’: ti,ab,kw<br>AND<br>‘cancer’: ti,ab,kw OR ‘neoplasia’: ti,ab,kw OR ‘neoplasm’: ti,ab,kw OR ‘tumor’: ti,ab,kw OR ‘malignancy’: ti,ab,kw<br>AND<br>‘low molecular weight heparin’: ti,ab,kw OR ‘LMWH’: ti,ab,kw OR ‘enoxaparin’: ti,ab,kw OR ‘dalteparin’: ti,ab,kw OR ‘nadroparin’: ti,ab,kw	562
Cochrane Library	MeSH descriptor: [dabigatran] OR dabigatran: ti,ab,kw OR Pradaxa: ti,ab,kw OR MeSH descriptor: [rivaroxaban] OR rivaroxaban: ti,ab,kw OR Xarelto: ti,ab,kw OR MeSH descriptor: [apixaban] OR apixaban: ti,ab,kw OR Eliquis: ti,ab,kw OR MeSH descriptor: [edoxaban] OR edoxaban: ti,ab,kw OR Savaysa: ti,ab,kw OR MeSH descriptor: [betrixaban] OR betrixaban: ti,ab,kw OR Bevyxxa: ti,ab,kw OR Non-vitamin K antagonist oral anticoagulants: ti,ab,kw OR NOACs: ti,ab,kw OR direct oral	91

	anticoagulants: ti,ab,kw OR DOACs: ti,ab,kw OR novel oral anticoagulants: ti,ab,kw OR new oral anticoagulants: ti,ab,kw OR factor Xa inhibitors: ti,ab,kw OR factor IIa inhibitors: ti,ab,kw<br>AND<br>MeSH descriptor: [cancer] OR cancer: ti,ab,kw OR neoplasia: ti,ab,kw OR neoplasm: ti,ab,kw OR tumor: ti,ab,kw OR malignancy: ti,ab,kw<br>AND MeSH descriptor: [low molecular weight heparin] OR low molecular weight heparin: ti,ab,kw OR LMWH: ti,ab,kw OR enoxaparin: ti,ab,kw OR dalteparin: ti,ab,kw OR nadroparin: ti,ab,kw	
Clinicaltrail.gov		5
Overall		946
Duplication		134

Supplementary Table 2. Definition of major bleeding in each study

Study	Definition of major bleeding
Hokusai VTE Cancer (2018)	Major bleeding was defined as overt bleeding that was associated with a decrease in the hemoglobin level of 2 g per deciliter or more, led to a transfusion of 2 or more units of blood, occurred in a critical site, or contributed to death.
SELECT-D (2018)	Acute, clinically overt bleeding accompanied by one or more of the following findings: a decrease in the hemoglobin level of > 20 g/L over a 24-hour period, transfusion of two or more units of packed red cells, bleeding at a critical site (including intracranial, intraspinal, intraocular, pericardial, or retroperitoneal bleeding), or fatal bleeding.
ADAM VTE (2019)	Major bleeding was defined as overt bleeding plus a hemoglobin decrease of ≥2 g/dL; or transfusion of ≥2 units of packed red blood cells; or intracranial, intraspinal/epidural, intraocular, retroperitoneal, pericardial, intraarticular, intramuscular with compartment syndrome, or fatal bleeding.
Caravaggio (2020)	Major bleeding was defined as acute clinically overt bleeding associated with one or more of the following: a decrease in the hemoglobin level of at least 2 g per deciliter, a transfusion of 2 or more units of red cells, bleeding occurring at a critical site (intracranial, intraspinal, intraocular, pericardial, intraarticular, intramuscular with compartment syndrome, or retroperitoneal), bleeding resulting in surgical intervention, or fatal bleeding, all occurring during the trial-drug period through 72 hours after the last dose was administered.
